# Inebriates

**Published:** 1892-05-07

**Authors:** 


### Passing Topics.

### INEBRIATES.

The promise of the Home Secretary to appoint a Select Committee to deal with matters affecting those unhappy creatures of whom society has agreed to discourse, somewhat euphoniously, as "inebriates," but who are more truthfully described as "habitual drunkards," is a welcome one. There can be no two opinions as to the need of some easily adopted means of imposing legitimate restraint upon the actions of people who are as often as not a terror to themselves and a pest to their friends and the community.

The spectacle of men and women painfully conscious of delinquency but utterly unable to summon a sufficiency of moral force to enable them to interpose an obstacle of any sort in the way of its indulgence is a piteous one, and there are probably few sinners who repent of their evil courses with greater, if intermittent, regularity than the victims of the craving which is so easily acquired but so hard to be quit of when once aroused. In other words no one is more conscious of his degradation than the inebriate in his lucid intervals, and it would almost seem as though his falling away is in the ratio of the vehemence of his self reproach and promise of amendment.

A proposition, of which the truth is tolerably obvious, is that a drunkard may be a worse member of society than the actual criminal. The consequences of his lapses are nearly always far-reaching, and as experience has only too abundantly shown, they may be prolific of crime. It is reasonable to urge that there are adequate grounds for instituting some better protection both for the victim and for society than is afforded in the provision of certain homes wherein the inebriate may undergo a period of voluntary incarceration at his own expense. There are not many sinners who are willing to surrender liberty whether for their own or their country's good, and whatever the depth of their repentance when the mood is upon them, it is hardly likely that drunkards—the very type and example of infirmity — should exhibit a sufficient tenacity of purpose, even supposing their means always enabled them to pay the cost of their conversion.

A little gentle compulsion and a consequent removal of pecuniary disability, is what we may hope the public conscience will ultimately cause to be applied. For our own part we do not fear that too drastic measures will be taken. We only regret that there is no possibility of going a little further, and of extending legislation to the case of the excessive drinker who never commits the weakness of getting drunk, but is none the less the arch-enemy of reason, sobriety, and peace. We observe that women furnish more than their proportion of offenders among the habitual drunkards. Possibly, this is because as a rule a woman may be more easily and visibly overcome. It is not unlikely that the men would somewhat redress the balance if we were enabled to take into account those frequent and terrible examples of inebriates whose excesses never show upon the surface, and who, even when saturated with drink, always contrive to walk straight, although it be to perdition and with their families upon their backs.

Agricola.

